# Is There a Role for Diabetes-Specific Nutrition Formulas as Meal Replacements in Type 2 Diabetes?

**DOI:** 10.3389/fendo.2022.874968

**Published:** 2022-04-29

**Authors:** Jarvis C. Noronha, Jeffrey I. Mechanick

**Affiliations:** ^1^ Toronto 3D (Diet, Digestive Tract and Disease) Knowledge Synthesis and Clinical Trials Unit, Clinical Nutrition and Risk Factor Modification Centre, St. Michael’s Hospital, Toronto, ON, Canada; ^2^ School of Medicine, Faculty of Medicine, The University of Queensland, Brisbane, QLD, Australia; ^3^ Marie-Josée and Henry R. Kravis Center for Cardiovascular Health at Mount Sinai Heart, and Division of Endocrinology, Diabetes and Bone Disease, Icahn School of Medicine at Mount Sinai, New York, NY, United States

**Keywords:** meal replacement, glycemic response, glycemic control, diabetes, clinical practice

## Abstract

Nutrition therapy plays an integral role in the prevention and management of patients with type 2 diabetes (T2D). A potential strategy is the utilization of diabetes-specific nutrition formulas (DSNFs) as meal replacements. In this article, we distinguish DSNFs from standard nutrition formulas, review the clinical data examining the effectiveness of DSNFs, and propose an evidence-based algorithm for incorporating DSNFs as part of nutrition therapy in T2D. DSNFs contain slowly-digestible carbohydrates, healthy fats (e.g., monounsaturated fatty acids), and specific micronutrients, which provide added benefits over standard nutrition formulas. In short- and long-term clinical trials, DSNFs demonstrate improvements in postprandial glycemic responses translating into sustainable benefits in long-term glycemic control (e.g., hemoglobin A1c and glycemic variability) and various cardiometabolic outcomes. To facilitate the delivery of DSNFs in a clinical setting, the transcultural diabetes nutrition algorithm can be utilized based on body weight (underweight, normal weight, or overweight) and level of glycemic control (controlled or uncontrolled).

## Introduction

Type 2 diabetes (T2D) is a well-established risk factor for cardiovascular disease, the leading cause of death in the world (1–3). Individuals with T2D are also at a significant risk for developing microvascular complications including, retinopathy, nephropathy and neuropathy. The International Diabetes Federation estimates that 643 million people or 11.3% of the global population will have diabetes by 2030. If these trends continue, this estimate is predicted to rise to 783 million or 12.2% of the global population by 2045 (4). Effective strategies are urgently needed to slow the burden of disease in this burgeoning population.

Nutrition therapy plays an integral role in the prevention and treatment of T2D. Core principles of nutrition and T2D include (1): individualized medical nutrition therapy (provided by a registered dietitian nutritionist whenever possible) that achieves healthy body composition, eating patterns, and macronutrient distributions based on preventative/therapeutic goals (with an emphasis on fiber, pulses, unprocessed whole foods, healthy fats, and limits on added sugars, refined grains, and alcohol); (2) recommendation of dietary supplements and nutraceuticals only when they are shown to have proven benefits that outweigh risks; and (3) synchronization of carbohydrate with insulin or secretagogue (e.g., sulfonylureas and glinides) therapy (5). In addition, clinical practice guidelines by the American Association of Clinical Endocrinology (formerly known as American Association of Clinical Endocrinologists), American Diabetes Association (ADA), Diabetes Canada (formerly known as Canadian Diabetes Association), and the European Association for the Study of Diabetes (EASD) recommend consumption of a low glycemic index (GI) diet that is high in fibre and monounsaturated fatty acids as part of healthy eating in T2D (6, 7).

Micronutrient deficiency states are common in T2D and should also be addressed: prevalence rates can range from 58-63% for vitamin B6 (pyridoxine), 13-55% for vitamin C, 85-91% for vitamin D, and 19% for zinc (8). These deficiency states are associated with increased oxidative stress, inflammation, and immune abnormalities (9). In T2D, nutritional deficiencies are associated with the progression of β-cell dysfunction, to loss of β-cell mass, and then to insulin signaling impairment and hyperinsulinemia (9). Nevertheless, as with any nutritional epidemiological finding and clinical correlate, the presence of a micronutrient deficiency (e.g., vitamin D deficiency) does not directly infer that clinically significant outcomes (e.g., improved glycemic control) will result with an intervention (e.g., vitamin D supplementation).

Notwithstanding the above caveat, diabetes-specific nutrition formulas (DSNFs) provide an opportunity to deliver essential macronutrients and micronutrients that align with clinical practice guideline recommendations for nutrition therapy in T2D. In this article, the role of DSNFs in T2D will be clarified based on the extant scientific literature. Specifically, DSNFs will be distinguished from standard nutrition formulas, clinical data on the effectiveness of DSNFs critically assessed, and an evidence-based algorithm for incorporating DSNFs as part of nutrition therapy in T2D proposed.

## Diabetes-Specific Nutrition Formulas Vs. Standard Formulas

DSNFs have a defined macronutrient and micronutrient composition to manage dysglycemia, malnutrition, and other cardiometabolic risk factors. These formulas contain modified carbohydrates that have a low GI and complementary dietary recommendations for patients with T2D. In addition, DSNFs generally contain fiber, unsaturated fatty acids, proteins, vitamins and minerals in palatable and calorie-controlled portions. These formulas are typically used as iso- or hypocaloric meal/snack replacements, hypercaloric supplementation for malnourished patients, enteral nutrition support, and in the context of very-low-calorie diets, as determined by clinical circumstances and the discretion of prescribing healthcare professionals. The macronutrient distribution (carbohydrates:fats:proteins) of DSNFs [37–55%:30–45%:15–19%, respectively and averaged from four common formulas (10–13)] compare well with distributions from various eating patterns, such as the Mediterranean Diet (40–50%:35–40%:15–20%, respectively) and clinical practice guidelines by professional societies (e.g., ADA: 45%:20–35%:15–20%, respectively) (9). In contrast, standard formulas may be high in rapidly digested carbohydrates (high GI), with varying fat content, and therefore compromising glycemic control.

## Review of Clinical Data Examining the Use of Diabetes-Specific Nutritional Formulas

The role of DSNFs in nutrition management of patients with T2D with respect to glycemic control and cardiometabolic risks can be clarified through a critical evaluation of the scientific data (**Table 1**).

**Table 1:** Effect of Diabetes-Specific Nutrition Formulas on Glycemic and Cardiometabolic Endpoints in Patients with Type 2 Diabetes.

Study [reference]	Design, Population	Intervention	Control	Main Results
Lansink et al. (12)	Parallel, RCT, 44 T2D patients	2x200mL of DSNF (Diasip®) for 4 weeks (n=22; 9M, 13F)	2x200mL isocaloric standard formula for 4 weeks (n=22; 16M, 6F)	↓PPGR in DSNF group at visit 1 (Day 1) and after 4 weeks use vs. control group
Laksir et al. (14)	Crossover, RCT, 19 T2D patients who were malnourished or at risk of malnourishment	1x200mL DSNF (Fortimel DiaCare) 2x100mL DSNF (Fortimel DiaCare)	1x200mL standard formula (Fortimel Extra)	↓PPGR and iCmax of glucose after DSNF consumption (1x200mL) vs. standard formula (1x200mL) ↓PPGR and PPIR after 2 half servings of DSNF (2x100mL) vs. full serving of DSNF (1x200mL)
Angarita Dávila et al. (15)	Crossover, RCT, 16 T2D patients	DSNF with resistant maltodextrin and sucromalt (GS; Glucerna SR®) DSNF with lactose, isomaltulose and resistant starch (DI; Diasip®)	Non-DSNF (ET; Ensure®)	↓PGR, PPIR and GIP response in GS and DI group vs. ET ↑GLP-1 response in GS group vs. ET and DI
Gulati et al. (16)	Crossover, RCT, 40 T2D patients	DSNF (Nutren® Diabetes)	Isocaloric meal (Cornflakes and milk)	↓PPGR and PPIR in DSNF group vs. isocaloric meal group
Mottalib et al. (17)	Crossover, RCT, 22 OW/OB patients with T2D	DSNF (Glucerna; GL) DSNF (Ultra Glucose Control; UGC)	Isocaloric meal (Oatmeal; OM)	↓PPGR and ↑ GLP-1 response after GL and UGC vs. OM
Mustad et al. (18)	Parallel, RCT, 81 T2D patients on oral anti-diabetes medications	Participants consumed DSNF (Glucerna Hunger Smart) at breakfast and as a mid-afternoon snack (DNSF Bkfst/AS; n=24) Participants consumed DSNF (Glucerna Hunger Smart) at breakfast and as a prebed snack (DNSF Bkfst/PBS; n=25)	Participants consumed no study product (SSD; n=32)	↓Positive AUC and ↓ adjusted peak value for glucose in DNSF Bkfst/AS group compared to SSD group ↓Nocturnal glycemic variability during the intervention phase compared with baseline phase in DSNF Bkfst/AS group
Chee et al. (19)	Parallel, RCT, 230 OW/OB patients with T2D	tDNA plan incorporating DSNFs with motivational interviewing for 6 months (tDNA-MI) tDNA plan incorporating DSNFs with conventional counseling for 6 months (tDNA-CC)	Usual care (UC) for 6 months	↓Body weight, HbA1c, and SBP in tDNA-MI and tDNA-CC groups vs. UC group at 6 months ↓FPG in tDNA-MI group vs. UC group at 6 months
Look AHEAD Group 2013 (20)	5145 OW/OB patients with T2D	Intensive lifestyle intervention (ILI) incorporating various MRs including DSNFs	Diabetes support and education (DSE) control group	↓Body weight and ↓ HbA1c in ILI group compared with control group throughout study period (median follow-up, 9.6 years)
Elia et al. (21)	SRMA, 23 studies, n=784 patients with T2D	DSNFs (oral or tube feeds)	Standard formulas	↓Postprandial rise in blood glucose, peak blood glucose, and glucose AUC; reduced requirement for insulin
Ojo et al. (22)	SRMA, 5 studies, n=270 patients with T2D	DSNFs (oral or tube feeds)	Standard formulas	↓FBG and HbA1c, and ↑HDL-c in DSF groups compared with standard formulas groups
Sanz-Paris et al. (23)	SRMA, 18 studies, n=845 patients with T1D/T2D	DSNFs high in MUFAs (oral or tube feeds)	Standard formulas	↓Postprandial glucose peak, incremental glucose response, glucose variability, HbA1c change from baseline, mean administered insulin dose, mean blood triglycerides and ↑ mean blood HDL

AUC, area under the curve; DSNF, diabetes-specific nutrition formula; FBG, fasting blood glucose; FPG, fasting plasma glucose; F, female; GIP, gastric inhibitory peptide; GLP-1, glucagon-like peptide-1; HbA1c, hemoglobin A1c; HDL, high-density lipoprotein; iCmax, incremental maximum concentration; M, male; OW/OB, overweight/obese; PPGR, postprandial glucose response; PPIR, postprandial insulin response; SBP, systolic blood pressure; tDNA, trans-cultural diabetes nutrition algorithm; T1D, type 1 diabetes; T2D, type 2 diabetes.

“down arrow” = reduced/lower.

“up arrow” = increased/higher.

*DSNFs vs. standard formulas*: there are demonstrable benefits of DSNFs on acute glucose tolerance when compared with standard nutrition formulas (12, 14, 15).

In a randomized, controlled, double-blind, parallel-group study, 44 patients with T2D consumed 2 x 200 mL DSNF/day (n=22) or an isocaloric standard, fiber-containing control formula (n=22) for 4 weeks. Results showed that the four-hour postprandial glucose response was significantly lower after consumption of the DSNF, compared with the standard formula group, at baseline and remained significantly lower after the 4-week intervention period, suggesting superior PPG control by DSNF (12). There was high compliance (>90%) in both groups. Both products were equally well tolerated, with a low incidence and mild intensity of reported symptoms. This study reported no serious adverse events and no dropouts for product-related reasons (12).

A separate study examined the effect of DSNF serving size in patients with T2D and malnutrition (or at-risk for malnutrition) (14). In this double blind, crossover study, 20 patients randomly received the DSNF (1 x 200mL) and the standard nutritional supplement control (1 x 200mL) on two separate days (visit 1 and 2), separated by a washout of 3-14 days. After a second wash-out period after visit 2, 17/20 patients completed an open label third study day (visit 3) where patients consumed the DSNF in serving sizes of 100 mL (2 x 100mL), of which the first serving was at breakfast and the second serving was 2 hours later. The 4-hour postprandial glucose response was lower after intake of the DSNF (1 x 200mL) compared with the control (1 x 200mL), with no difference in the insulin response. Additionally, the 4-hour postprandial glucose and insulin response was significantly lower after oral intake of two half servings of DSNF (2 x 100mL) compared with one full serving of DSNF (1 x 200mL). All patients were reported to have consumed the DSNF or control within the allocated time (10mins) and were 100% compliant for each dosing during intake. Adverse events were reported, however, the authors concluded that none of them were considered to be related to consumption of the study products and did not qualify as a safety signal. These data suggest that the use of a DSNF is preferred for patients with T2D in need of nutritional support and that splitting oral intake of DSNF into two half servings 2 hours apart may provide additional advantages in blunting postprandial glycemic and insulinemic excursions (14).

Lastly, a study which looked at more specialized DSNFs containing isomaltulose and sucromalt in 16 patients with T2D found lower postprandial glucose, insulin, and gastric inhibitory polypeptide responses with higher glucagon-like peptide-1 (GLP-1) levels, and decreased subjective appetite responses compared with standard formula (15). Seven patients withdrew from the study (initial sample: 23 patients): four withdrew due to introduction of medical/lifestyle therapy and three were due to voluntary withdrawal. The study products were well-tolerated with no patients reporting nausea, dizziness or vomiting after consumption.

*DSNFs vs. standard test meals*: there are demonstrable advantages of DSNFs over standard test meals in acute clinical trials (16, 17).

In an open-label, randomized, crossover study, 40 patients with T2D were randomly assigned to receive either a DSNF or an isocaloric meal (cornflakes and milk) after a run-in period. The DSNF group showed decreased post-meal blood glucose and insulin excursion as compared with the isocaloric meal comparator group. The authors concluded that the DSNF was well-tolerated and may be a suitable option for patients with T2D in need of nutritional support (16).

In a separate study, two DSNFs were compared with oatmeal on glucose, insulin, GLP-1, free fatty acid (FFA) and triglyceride (TG) responses. Twenty-five patients with T2D were enrolled, of which 22 patients completed all study visits. The three drop-outs were indicated to be for personal reasons. Both DSNFs resulted in lower postprandial glucose response and higher postprandial GLP-1 responses, compared with oatmeal, with no differences in insulin, FFA, and TG levels (17).

A recent pilot study evaluated the impact of DSNF used twice daily (either at breakfast and afternoon snack, or at breakfast and pre-bed snack) by patients with T2D (18). After a baseline period where study participants consumed their habitual self-selected diets for six days, patients with T2D were randomized into three groups: (1) self-selected diet group consumed no study product; (2) DSNF breakfast/afternoon snack group consumed one DSNF as a breakfast meal replacement and a second as a mid-afternoon snack replacement; and (3) DSNF breakfast/pre-bed snack group consumed one DSNF as a breakfast meal replacement and a second as a pre-bed snack replacement. Glycemic response was assessed by continuous glucose monitoring. One-hundred and twenty-five patients were randomized of which 116 completed the study. A total of 81 patients who were evaluable (>80% adherent to study instructions) were analyzed (DSNF breakfast/afternoon snack group, N=24; DSNF breakfast/pre-bed snack group, N=25; self-selected diet group, n=32). Patients were well-compliant during the intervention phase (~6 days) consuming ~2 servings of DNSF per day. Overall, the DSNF breakfast/afternoon snack group demonstrated greater reductions in positive area under the curve (AUC) and adjusted peak value for blood glucose when compared to the self-selected diet group. Interestingly, the DSNF breakfast/afternoon snack group also had greater reductions in nocturnal glycemic variability, reported less cravings for starchy meals/sides, and exhibited increased confidence in selecting foods to control their diabetes when compared to baseline measurements prior to interventions. These data suggest that use of DNSFs to replace breakfast and as an afternoon snack can improve glycemic control and behavioral factors associated with management of T2D.

*DNSFs in longer-term studies*: sustainable reductions in glycemic control have also been observed with DSNF use (19, 20).

In a study conducted in Malaysia which implemented the transcultural diabetes nutrition algorithm (tDNA), patients with T2D were randomized to one of three interventions for 6 months: (1) tDNA with motivational interviewing, (2) tDNA with conventional counseling, or (3) usual care. The tDNA intervention consisted of a structured low-calorie meal plan, DSNFs, and increased physical activity. All patients were also followed up at 12 months. Patient retention rates were: 51/58 (88%) in the tDNA with motivational interviewing group, 40/57 (70%) in tDNA with conventional counseling group, and 98/115 (85%) in the usual care group. At 6 months, hemoglobin A1c (HbA1c) decreased on average by 1.1% in patients incorporating tDNA with motivational interviewing and 0.5% in patients incorporating tDNA with conventional counseling, with no significant changes in patients under usual care. Body weight also decreased significantly in patients incorporating tDNA with motivational interviewing (~7kg) and in patients incorporating tDNA with conventional counseling (~5kg), with no significant changes in patients under usual care. At 1 year, weight loss and reductions in HbA1c were only maintained in patients incorporating tDNA with motivational interviewing (19). In a follow-up study (24), eating self-efficacy was assessed using a locally validated Weight Efficacy Lifestyle (WEL) questionnaire. Eating self-efficacy is the confidence in an individual’s own ability to perform specific tasks to attain certain goals in challenging situations. Overall, eating self-efficacy improved in patients who maintained their weight loss and glycemic control following the tDNA approach utilizing DSNFs and the improvement was further enhanced with motivational interviewing.

In the large, multi-centre, Look AHEAD Trial (20, 25, 26), an intensive lifestyle intervention group, which incorporated various meal replacements including DSNFs, was compared to a diabetes support and education group. Although widespread significant comparative differences were seen in the first year, participants in the intensive lifestyle intervention group retained greater improvements than participants in the diabetes support and education group at 4 years in % weight loss (-6.15% vs. -0.88%, p < 0.001), HbA1c (-0.36% vs. -0.09%, p < 0.001), systolic blood pressure (-5.33 vs. -2.97 mmHg, p < 0.001), diastolic blood pressure (-2.92 vs. -2.48 mmHg, p=0.01), and high-density lipoprotein (HDL)-cholesterol (3.67 vs. 1.97 mg/dL, p < 0.001) (25). At year 8, clinically meaningful weight loss of ≥5% was still observed in 50.3% of patients in the intensive lifestyle intervention group (26).

*Evidence from systematic reviews and meta-analyses*: several systematic reviews and meta-analyses have also been conducted to examine the totality of the evidence in relation to the effectiveness of DSNFs.

In 2005, Elia et al. (21) identified 20 studies that compared the short- and long-term use of DSNFs with standard formulas on glycemic control in patients with type 1 diabetes or T2D. They found that DSNFs, when compared with standard formulas, resulted in significantly reduced postprandial rise in blood glucose, peak blood glucose concentration, and glucose AUC (by ~35%) with no significant effect on HDL, total cholesterol, or triglyceride concentrations. Reduced insulin requirement and fewer complications with DSNFs were also observed in individual studies when compared with standard nutritional formulas.

A separate systematic review and meta-analysis conducted by Ojo et al. (22) in 2019 focused on evaluating the effectiveness of DSNFs versus standard formulas on glycemic control in patients with T2D. Meta-analysis of 5 studies showed that DSNFs resulted in significantly lower fasting blood glucose levels (by 1.15 mmol [-2.07, -0.23]) and HbA1c levels (by 0.67% [-1.14, -0.21]) with no effect on total cholesterol, low-density lipoprotein cholesterol, and triglycerides when compared to standard formulas. They also found slightly greater HDL-c levels (by 0.09 mmol/L [0.00, 0.18]) in DSNFs groups compared with standard formula groups.

Lastly, a recent systematic review and meta-analysis conducted by Sanz-Paris et al. (23) compared the effects of DSNFs with a high content of monounsaturated fatty acids with standard formulas on outcomes of glycemic control, lipid metabolism, and tolerance. Eighteen studies involving 845 patients were included. This meta-analysis showed that DSNFs with a high content of monounsaturated fatty acids significantly decreased peak postprandial glucose, incremental glucose response, mean blood glucose, glucose variability, HbA1c, AUC plasma insulin, mean administered insulin dose, and mean blood total triglycerides, as well as significantly increased HDL-c (23). Analysis of gastrointestinal adverse events reported by authors of the included studies revealed no significant differences among treatments.

## Clinical Approach for Diabetes-Specific Nutrition Formulas

In 2010, the tDNA was developed as a tool to facilitate the delivery of nutrition therapy to patients with prediabetes and T2D in a variety of cultures and geographic locations. This included guidance on how many DSNFs patients should consume based on their body weight (underweight, normal weight or overweight) and level of glycemic control (uncontrolled or controlled) (**Table 2**). For example, patients with T2D and overweight/obesity are recommended to consume 2 to 3 DSNFs per day, as meal and/or snack replacements, as part of a reduced calorie meal plan. In contrast, patients with uncontrolled T2D (i.e., HbA1c> 7%) are recommended to consume 1 to 2 DSNFs per day, as meal and/or snack replacements, as part of a lower GI meal plan (27).

**Table 2:** Evidence-Based Algorithm for the use of Diabetes-Specific Nutrition Formulas in Prediabetes, Type 1 Diabetes and Type 2 Diabetes.

Overweight/obese individuals	Use 2 to 3 diabetes-specific nutrition formulas^a^ as part of a reduced calorie meal plan, as a calorie replacement for meal, partial meal, or snack (grade C; LOE 3) Calorie goals (from diabetes-specific nutrition formulas and from other healthy dietary sources): <250lb = 1200 to 1500 calories >250lb = 1500 to 1800 calories	
Normal weight individuals	Uncontrolled diabetes HbA1c>7%	1 to 2 diabetes-specific nutrition formulas per day to be incorporated into a meal plan, as a calorie replacement for meal, partial meal, or snack (grade D; LOE 4)
	Controlled diabetes HbA1c<7%	Use of diabetes-specific nutrition formulas should be based on clinical judgement and individual assessment^b^ (grade D; LOE 4)
Underweight individuals	Use of diabetes-specific nutrition supplements^c^ 1 to 3 units/d per clinical judgement based on desired rate of weight gain and clinical tolerance (grade D; LOE 4)	

Adapted from (27).

LOE 1: data defined as conclusive results from prospective, randomized controlled trials that have large subject populations representative of the target population and results that are easily generalized to the target population. Data also include results from meta-analyses of randomized controlled trials, results from multicenter trials, and “all or none” evidence; LOE 2: data include conclusive results from individual randomized controlled trials that have limited subject numbers or target population representation; LOE 3: data include all other conclusive clinical findings from nonrandomized studies, studies without controls, and nonexperimental or observational studies. These data may require interpretation and, by themselves, are not compelling; LOE 4: data are defined as information based solely on experience or expert opinion and are not necessarily substantiated by any conclusive scientific data. Frequently, only LOE 4 data are available.

a Diabetes-specific nutrition formulas are nutritional products used as replacement for meals, partial meals, or snacks to replace calories in the diet.

b Individuals who may have muscle mass and/or function loss and/or micronutrient deficiency may benefit from diabetes-specific nutrition supplements. Individuals who need support with weight maintenance and/or a healthy meal plan could benefit from diabetes-specific nutrition formulas.

c Diabetes-specific nutrition supplements are complete and balanced nutritional products used in addition to a typical meal plan, to help promote increased nutritional intake.

While the use of DSNFs in each population group are evidence-based, the specific dosing required has not been validated and therefore, expert opinion by international thought leaders was required to determine the number of unit doses for each clinical scenario. Implementation and adherence to the tDNA will also vary depending on cultural, psychological, ethnic, nutritional, and lifestyle factors, as well as the economic and social environment. A local task force may be required to detail the factors that need to be considered in the adaptation of the algorithm to specific regions, as was done in the Venezuelan adaptation of tDNA (28).

## Future Directions

Despite the current evidence of the role of DSNFs in T2D, significant research, knowledge and practice gaps remain. There is a need for additional studies to elucidate the mechanism of action, along with larger randomized controlled trials, more relevant clinical studies that reflect real-world scenarios, and economic/cost studies to improve our certainty in the utility of DNSFs for patients with T2D. Additionally, knowledge gaps among various healthcare professionals (HCPs) such as, nurses, registered dietitians, and physicians, need to be addressed through education and training, while subsequently targeting practice gaps which enable HCPs in implementing DSNFs in the clinical setting (e.g., through the use of validated clinical practice algorithms).

## Conclusions

The current evidence indicates that there is a role for DSNFs as meal/snack replacements in nutrition therapy for T2D. DNSFs have demonstrated reduced glycemic and insulin excursions when compared with standard formulas and some commonly consumed foods. These acute benefits have also translated into meaningful reductions in long-term glycemic control along with improvements in various cardiometabolic endpoints. The tDNA is an evidenced-based approach that can be used to facilitate the delivery of DSNFs to patients with T2D in the clinical setting.

## Data Availability Statement

The original contributions presented in the study are included in the article/supplementary material. Further inquiries can be directed to the corresponding author.

## Author Contributions

JN drafted the manuscript. JM critically reviewed the manuscript for important intellectual content. Both authors reviewed and approved the final manuscript.

## Funding

This work was supported by the Toronto 3D Knowledge Synthesis and Clinical Trials foundation.

## Conflict of Interest

JN has worked as a clinical research coordinator at INQUIS Clinical Research. He has also received research support from Glycemia Consulting Inc. JM has received honoraria for lectures and program development from Abbott Nutrition and serves on the Advisory Boards of Aveta.Life, L-Nutra, and Twin Health.

## Publisher’s Note

All claims expressed in this article are solely those of the authors and do not necessarily represent those of their affiliated organizations, or those of the publisher, the editors and the reviewers. Any product that may be evaluated in this article, or claim that may be made by its manufacturer, is not guaranteed or endorsed by the publisher.
